# Early breast cancer detection in CT scans using convolutional neural bidirectional feature pyramid network

**DOI:** 10.7717/peerj-cs.2994

**Published:** 2025-07-03

**Authors:** Tahani Jaser Alahmadi, Adeel Ahmed, Amjad Rehman, Abeer Rashad Mirdad, Bayan Al Ghofaily, Shehryar Ali

**Affiliations:** 1 Department of Information Systems, College of Computer and Information Sciences, Princess Nourah bint Abdulrahman University, Riyadh, Saudi Arabia; 2 Department of Information Technology, The University of Haripur, Haripur, Pakistan; 3 Artificial Intelligence & Data Analytics Lab (AIDA) CCIS, Prince Sultan University, Riyadh, Saudi Arabia

**Keywords:** Convolutional neural network, Breast cancer, Computed tomography, Bidirectional feature pyramid network, Multi-scale features, Tumor localization

## Abstract

Breast cancer is one of the leading causes of death among women worldwide. Early detection plays a crucial role in reducing mortality rates. While mammography is a widely used diagnostic tool, computed tomography (CT) scans are increasingly being explored for detecting breast cancer due to their high-resolution imaging and ability to visualize tissue in 3D. Despite the potential of CT scans in visualizing breast tissue in 3D with high resolution, extracting meaningful patterns from these scans is difficult due to the complex and nonlinear nature of the tissue features. The challenge lies in developing computational methods that can accurately detect and localize breast cancer lesions, especially when the tumors vary in size, shape, and density. In this article, we proposed a framework called convolutional neural bidirectional feature pyramid network, which integrates multi-scale feature extraction and bidirectional feature fusion for breast cancer detection in CT scans. The proposed framework classified the images into diseased and non-diseased and then identified the infected region on breast tissue. Using convolutional neural networks, we defined several layers to classify the diseased and normal CT scan images. We collected data on breast CT scans taken from the radiology department, Ayub Teaching Hospital Abbottabad, Pakistan. We evaluated the model using a variety of classification metrics such as precision, recall, F1-measure, and average precision to determine its effectiveness in finding breast cancer lesions, and we found 96.11% accuracy. Our findings show that compared with current state-of-the-art methods, the proposed framework has satisfactory results in identifying breast cancer areas, and the proposed framework over the baselines has achieved a 1.71% improvement.

## Introduction

Despite various approaches for detection and prevention, the incidence of human illnesses has progressively risen due to individual or environmental factors. Among these illnesses, cancer is the most prevalent global affliction, characterized by abnormal cell growth that impacts different body areas. Cancer manifests in various forms, including breast, prostate, lung, skin, and pancreatic cancer, and remains a leading cause of death worldwide (Rajinikanth et al., 2021). Early detection is crucial, as undiagnosed breast cancer can be fatal. Advances in early diagnosis and treatment have significantly improved survival rates. Various imaging techniques are employed for breast cancer screening, including X-ray radiography, computed tomography (CT) scans, ultrasonography, magnetic resonance imaging (MRI), and thermal imaging, each offering unique advantages in detecting abnormalities.

Each modality employs unique methods and tools, yielding results influenced by various factors, making it advisable to combine multiple methods for result validation. Mammography, the most prevalent screening procedure for breast cancer, is often considered the benchmark, but it poses considerable risks to patients. Consequently, thermal imaging has garnered attention due to its lower risk profile than mammography (Singh & Singh, 2020). Despite not yet being the primary early breast cancer detection method, digital infrared thermal imaging has shown promise. While relying on skin lesions and temperature, it can complement mammography when used in conjunction, enabling radiologists to assess and diagnose breast cancer (Parisky et al., 2000) more accurately.

Artificial neural networks (ANNs) employ mathematical models and emulate the structure of mammalian neural systems on a smaller scale (LeCun, Bengio & Hinton, 2015; Yamashita et al., 2018). Whether object detection, image classification, or image segmentation, convolutional neural networks (CNNs) have demonstrated superior capabilities in automatically learning and extracting relevant image features (Bhojanapalli et al., 2021).

Transformer-based architectures have recently shown great potential as breast cancer detection tools, significantly improving traditional algorithms such as CNNs. Unlike CNNs, which are based on local feature extraction using fixed spatial extent of the kernel, transformers use self-attention mechanisms to capture arbitrary-length context across the whole image. This capability enables transformer models like Vision Transformers (ViTs) and Swin Transformers to examine complex patterns in the medical imaging data such as mammograms, and CT scans. Recent studies have shown that ViTs outperform CNNs in classifying breast cancer lesions since they can develop better feature extraction and understanding of spatial relation across various locations in an image (Dosovitskiy et al., 2020). Swin Transformer, which proposes hierarchical feature learning through shifted windows, also boosts tumor segmentation accuracy, assisting radiologists in obtaining accurate and early diagnoses (Liu et al., 2021). Hybrid architectures, combining CNNs and transformers, have improved breast cancer diagnosis even more by aligning the regional feature extraction performed by CNNs with the contextual features offered by transformers (Liu et al., 2021).

Traditional imaging methods, such as mammography, are widely used for breast cancer detection but suffer from drawbacks like radiation exposure, high false positive/negative rates, and challenges in detecting tumors in dense breast tissue. While CT scans offer high-resolution imaging, extracting meaningful patterns requires advanced computational techniques. Though effective in feature extraction, conventional CNN-based approaches struggle with detecting tumors of varying sizes due to their fixed receptive fields and inefficient multi-scale feature utilization, making them less robust for complex medical imaging tasks. To address this limitation, our study integrates bidirectional feature pyramid network (BiFPN) (Tan & Le, 2019; Redmon & Farhadi, 2018), which enhances feature fusion by leveraging multi-scale representations, enabling better tumor localization and improving detection accuracy. Unlike standard CNNs, BiFPN refines features bidirectionally, capturing both fine-grained details and high-level contextual information, thus providing a more reliable approach for breast cancer detection in CT scans.

### Contributions

In this article, our contributions are:

- We have developed a hybrid framework called convolutional neural bidirectional feature pyramid network (CNBiFPN), which integrates multi-scale feature extraction and bidirectional feature fusion for breast cancer detection in CT scans. This framework identifies the infected regions with cancerous cells over breast tissue and labelled them with bounding boxes around the regions of interest.
- Using convolutional neural networks, we defined several layers to classify the diseased and normal CT scan images.
- We have performed experiments over a real CT scan dataset collected from Ayub Teaching Hospital Complex Abbottabad, Pakistan.

### Rationale

Existing methods often rely on single-scale feature extraction, leading to suboptimal performance detecting malignancies with diverse shapes, sizes, and tissue densities (Xie et al., 2021). Furthermore, while CNN-based models have been widely used, they struggle with localization accuracy due to fixed receptive fields and limited ability to integrate global contextual information (Dosovitskiy et al., 2020). We proposed convolutional neural bidirectional feature pyramid network (CNN-BiFPN) framework with a feature pyramid convolution network with multi-scale feature extraction, which is very important in the research about breast cancer detection. Standard feature pyramid network (FPNs) tries to overcome these problems by combining multi-scale feature maps (Lin et al., 2017a). The BiFPN, a superior version of FPN that involves a bidirectional cross-scale characteristic assimilation with arranged weighted connections, guarantees that the relevant traits are conveyed effectively throughout the network (Tan, Pang & Le, 2020). The rationale behind choosing proposed CNN-BiFPN is its advanced ability to efficiently handle multi-scale features (important for detecting tumors of varying sizes in breast scans) and improve performance by leveraging bidirectional feature fusion. This allows the model to capture fine-grained details and high-level context, which is crucial for accurate lesion detection.

The rest of the article is structured as follows. “Related Work” describes the related work, “Proposed Framework” discusses the proposed approach, “Experimental Setup and Results” explains the experimental setup and results, and “Conclusion” discusses the conclusion.

## Related work

In recent years, many machine-learning techniques have been applied to analyze breast cancer imaging. One innovative approach combined convolutional neural networks and long short-term memory networks to classify over 7,000 histological slides. After extracting features, the proposed model fed them into softmax and support vector machine classifiers achieving an impressive 96% precision on low magnification images and 91% highest accuracy on high magnification, consistent with advances. Another study (Rashed & El Seoud, 2019) introduced a novel U-net inspired convolutional neural network designed for mammography datasets focusing on masses and calcifications. The network trained on nearly 700 mass images and over 600 calcification images, reserving over 200 and 150 respectively for testing, achieving a remarkable 94.31% accuracy. An additional innovative model, IRRCNN (Arslan, Yaşar & Çolak, 2019), was a convolutional neural network framework to classify breast cancer histopathology using the same dataset, achieving 91.4% accuracy during testing and demonstrating effectiveness. Furthermore, BC-DROID enables fully automated detection and classification in a single step using convolutional neural networks. Trained on over 10,000 complete mammograms, BC-DROID achieved 90% detection accuracy, 93.5% classification accuracy and 92.315% area under the curve.

A hybrid deep learning model named CRNN, described in Patil & Biradar (2021), was used to detect breast cancer from mammographic images. The process was to build a gray level cooccurrence matrix and a gray level run length matrix as feature inputs. Using a series of SELECT && operations, the diagnostic results derived by the classifier proved to be accurate in 90.59% of cases. Compared with traditional models, this accuracy is superior by far. With the rapid development of infrared camera technology, research on diagnosing and classifying breast cancer based on thermal images has also made significant progress. Many studies have used machine learning (ML) and deep learning (DL) algorithms to analyze this form of images.

A comprehensive analysis of 1,052 thermogram images was conducted at The Federal University of Pernambuco University Hospital in 2015 (Santana et al., 2018). Bayes network, naïve Bayes, support vector machine (SVM), J48 decision tree algorithm, random forest (RF), multi-layer perceptron, random forest tree, among these classifiers, MLP is a good prospect with accuracy rate of 73.38%, kappa index value reaches 0.6007, sensitivity 78% and specificity 88%. Furthermore, after reflecting changes using 10-fold cross-validation, there was a better kappa index value 0.6402 now at an improved accuracy rate 76.01%. Overall, system effectiveness improved from 83% to 84%.

To enhance the data classification with ALR and KCC for DBT-TU-JU and DMR-IR series, classifier algorithms artificial neural network (ANN), SVM, k-nearest neighbor (KNN) and decision trees (DT) were improved with features SSigFS, FStat, and STex (Gogoi et al., 2017). The SVM-RBF and ANN models scored a better accuracy rate of 84.29% on DVDTU-JU series, While ANN and SVM-linus are the best classifiers in k-fold using DSIE-IR based and picked 87.50% accuracy rate. Deep learning (DL) techniques, especially convolutional neural networks (CNNs), have also been employed to study thermogram images. In Ekici & Jawzal (2020), a CNN was trained on histopathology images and fine-tuned by Bayes optimization. Authors collected 1,116 images from the DMI data set, the method achieved a remarkable minimum error rate of 98.05%, which outshone earlier reports using different feature sets and browse classification on the same dataset.

In another work (Mishra et al., 2020), employed a large DCNN designed especially for breast cancer diagnosis from thermography images. To concisely formalise the results, 680 images from the Visual lab-IR data set converted to grayscale images were effectively segmented and categorized. The forecasting accuracy rate is 95.8%, which is better than (Mambou et al., 2018). A study in 2012 (Mookiah, Acharya & Ng, 2012) used decision trees and fuzzy classifiers with an accuracy 92.30%.

Tello-Mijares, Woo & Flores (2019) investigated a combination of generalized vector field (GVF) breast segmentation with CNN for breast cancer classification (Nahid, Mehrabi & Kong, 2018). In this study, 63 breast images from DMR-IR dataset were used, which were classified as Normal and Abnormal. With 2-fold cross-validation, the model delivered outstanding results, with accuracy, sensitivity, and specificity, all at an extraordinary 100% level. Importantly, their performance was higher than that of tree random forest (TRF), multi-layer perceptron (MLP), and Bayes network classifiers. For Sanyal, Jethanandani & Sarkar (2020) an attention mechanism (AM) was used to perform the categorization of high-resolution histological images from breast tissue with (CNN + bidirectional long short-term memory (BLSTM)). The model’s training was conducted on the ICIAR 2018 database. The experimental findings unveiled that the model attained an accuracy of 85.50% for classifying images into four classes and an even more remarkable accuracy of 96.25% for two classes. A comparative analysis was performed with and without the AM and against state-of-the-art methods. Notably, the model consistently outperformed other approaches with and without integrating the attention mechanism. In Deng et al. (2020), an enhancement was made to CNNs by incorporating an innovative SE-attention mechanism, which proved highly effective in classifying a collection of 18,157 mammograms. A new benchmarking dataset was curated for this purpose, and the model demonstrated superior performance compared to other studies, achieving an accuracy rate of 92.17%. To enhance performance on the BreakHis dataset, Zhang et al. (2019) proposed a classification framework for breast cancer tissue images based on ResNet integrated with a convolutional block attention module (CBAM). For improving performance on the BreakHis dataset, Zhang et al. (2019) designed a classification framework using RESNET integrated with CBAM to classify images of breast cancer tissue. The model is then used to classify the 7,909 images with significant increases to an accuracy of 92.6%, sensitivity of 94.7%, specificity of 88.9%, F1-score of 94.1%, and an AUC of 91.8% for 200× magnified images. Additionally, the proposed model outperformed ResNet50 and ResNet-50 with CBAM in terms of accuracy by a large margin at both the patient and image levels. Franceschini et al. (2023) introduced a deep learning method for tumor detection in a microwave tomography framework, yielding promising results in identifying small tumor masses. Gengtian, Bing & Guoyou (2023) applied the EfficientNet architecture for detecting and classifying breast cancer in mammography images, highlighting the effectiveness of deep learning in medical imaging. Additionally, deep learning models like CNN and Vgg16 for evaluating histopathology images have demonstrated great potential in improving both the accuracy and speed of breast cancer detection.

The hybrid model developed by Ametefe et al. (2025) connected deep transfer learning methods with U-Net segmentation to find breast cancer through ultrasound reviews. A main drawback of this approach is that it exclusively uses ultrasound images, which prevents direct application across other imaging systems. Al-Duais et al. (2025) discussed the development of a comparative approach of classical machine learning technologies like SVM, KNN, and RF appropriate for early breast cancer detection. Fontes et al. (2025) presented a new 3D CNN method designed for luminal A breast cancer phenotyping on CT and MRI images. Their model greatly improved the classification accuracy compared to traditional 2D CNNs by considering spatial features. Marinakis et al. (2025) discussed the precision challenge in a study on 3D residual networks for segmenting pulmonary nodules in low-dose CT scans whereas, initially targeted to lung cancer, which is also acceptable for detecting breast lesions. Their data generation with the patch helped the model to be much more sensitive to small lesions, a key requirement for early screening for breast cancer (Marinakis et al., 2025). A multi-modal fusion system composed of mammography, MRI and CT scan has been proposed by Lew et al. (2025) with the intentions of increasing breast cancer detection accuracy. Wang et al. (2025) participated in the RSNA Breast Cancer Detection competition and applied deep learning model on CT data to predict mammographic breast density. Although not a primary detection task, accurate density assessment is a strong indicator of cancer risk and their model provided an important auxiliary tool for risk stratification (Wang et al., 2025). Sun et al. (2025) proposed DL models based on chest CT images to differentiate benign and malignant breast masses and predict axillary lymph node metastasis. Their strategy offered a non-invasive substitute for biopsy, which had substantial potential for clinical use but also stressed the significant need for large-scale validation (Sun et al., 2025). Tong et al. (2025) introduced the integration of machine learning with panoramic photoacoustic computed tomography (PACT) for breast lesion analysis. Although primarily experimental, this technique offered an innovative alternative to conventional CT and showed that DL could improve lesion boundary detection and characterization (Tong et al., 2025).

Breast cancer detection through deep learning has been thoroughly investigated, with various studies concentrating on CNN-based frameworks, feature extraction methods, and segmentation techniques. While prior research has achieved notable advancements, significant gaps still need to be addressed. Despite their robust feature extraction abilities, conventional CNN architectures frequently face challenges in detecting tumors across multiple scales, particularly in intricate medical imaging contexts where tumors exhibit considerable variation in size, shape, and contrast. The work conducted by Lin et al. (2017a) introduced feature pyramid networks (FPNs) to tackle the issue of multi-dimension feature learning; however, these models experience limitations due to unidirectional feature propagation, resulting in suboptimal feature fusion. Likewise, alternative methods, including U-Net and Mask R-CNN, have been utilized for medical image segmentation but no dynamic feature reweighting (He et al., 2017; Ronneberger, Fischer & Brox, 2015).

The proposed framework incorporated the BiFPN with CNNs, facilitating effective multi-scale feature fusion through iterative bidirectional information flow. In contrast to conventional FPNs, BiFPN enhances feature selection *via* dynamic weighted connections, guaranteeing that only the most pertinent features play a role in the final classification and segmentation processes (Tan, Pang & Le, 2020). This pivotal improvement boosts tumor localization, classification precision, and reduces false positives, which are frequent limitations of current CNN-based models. Mughal et al. (2018) proposed a novel classification system to reduce breast tumor-related mortality among women. Their approach demonstrated enhanced diagnostic reliability using microscopy imaging techniques (Mughal et al., 2018). Elkorany et al. (2022) utilized SVM optimized by whale optimization algorithm (WOA) and dragonfly algorithm (DA) for breast cancer diagnosis. The dual-optimization technique enhanced classification performance significantly over standard SVM (Elkorany et al., 2022). Marie-Sainte et al. (2019) developed an improved prediction model for breast cancer diagnosis, survivability, and recurrence. The strategy combined data mining techniques with machine learning to forecast patient outcomes. Results showed that the model outperformed conventional methods in predictive accuracy and decision support (Marie-Sainte et al., 2019). Table 1 describes the literature review with drawbacks of the existing approaches.

**Table 1:** Literature review.

Reference	Approach used	Drawbacks	Advantage	Dataset used
Rashed & El Seoud (2019)	U-Net inspired CNN	Limited to mammography datasets only	94.31% accuracy on mass/calcification images	700 mass & 600 calcification mammograms
Platania et al. (2017)	BC-DROID (CNN)	One-step approach may reduce modular control	90% detection, 93.5% classification, 92.3% AUC	10,000 mammograms
Patil & Biradar (2021)	CRNN (CNN + RNN)	Complexity due to hybrid model	90.59% accuracy	Mammographic images
Santana et al. (2018)	Various ML classifiers (MLP best)	Moderate accuracy	MLP achieved 76.01%, better kappa index	1,052 thermograms
Gogoi et al. (2017)	ANN, SVM, KNN, DT	Dependent on hand-crafted features	87.5% accuracy	DBT-TU-JU & DMR-IR
Ekici & Jawzal (2020)	CNN + Bayes optimization	Manual tuning needed	98.05% minimum error	DMI dataset (1,116 histopathology images)
Mishra et al. (2020)	DCNN on thermography	Grayscale conversion required	95.8% accuracy	680 images from Visual lab-IR
Mookiah, Acharya & Ng (2012)	DT + Fuzzy classifier	Older model	92.3% accuracy	Not specified
Tello-Mijares, Woo & Flores (2019)	GVF + CNN	Small dataset	100% accuracy, specificity, sensitivity	63 images (DMR-IR)
Sanyal, Jethanandani & Sarkar (2020)	CNN + BLSTM + AM	High model complexity	96.25% (binary), 85.5% (4 classes)	ICIAR 2018
Deng et al. (2020)	SE-attention CNN	High resource demand	92.17% accuracy	18,157 mammograms
Zhang et al. (2019)	ResNet + CBAM	Limited to BreakHis dataset	92.6% accuracy, 94.7% sensitivity	7,909 images (BreakHis)
Franceschini et al. (2023)	DL in microwave tomography	Special imaging modality	Effective for small tumors	Not specified
Gengtian, Bing & Guoyou (2023)	EfficientNet on mammograms	Requires high computational resources	High accuracy	Not specified

## Proposed framework

**Figure 1:**
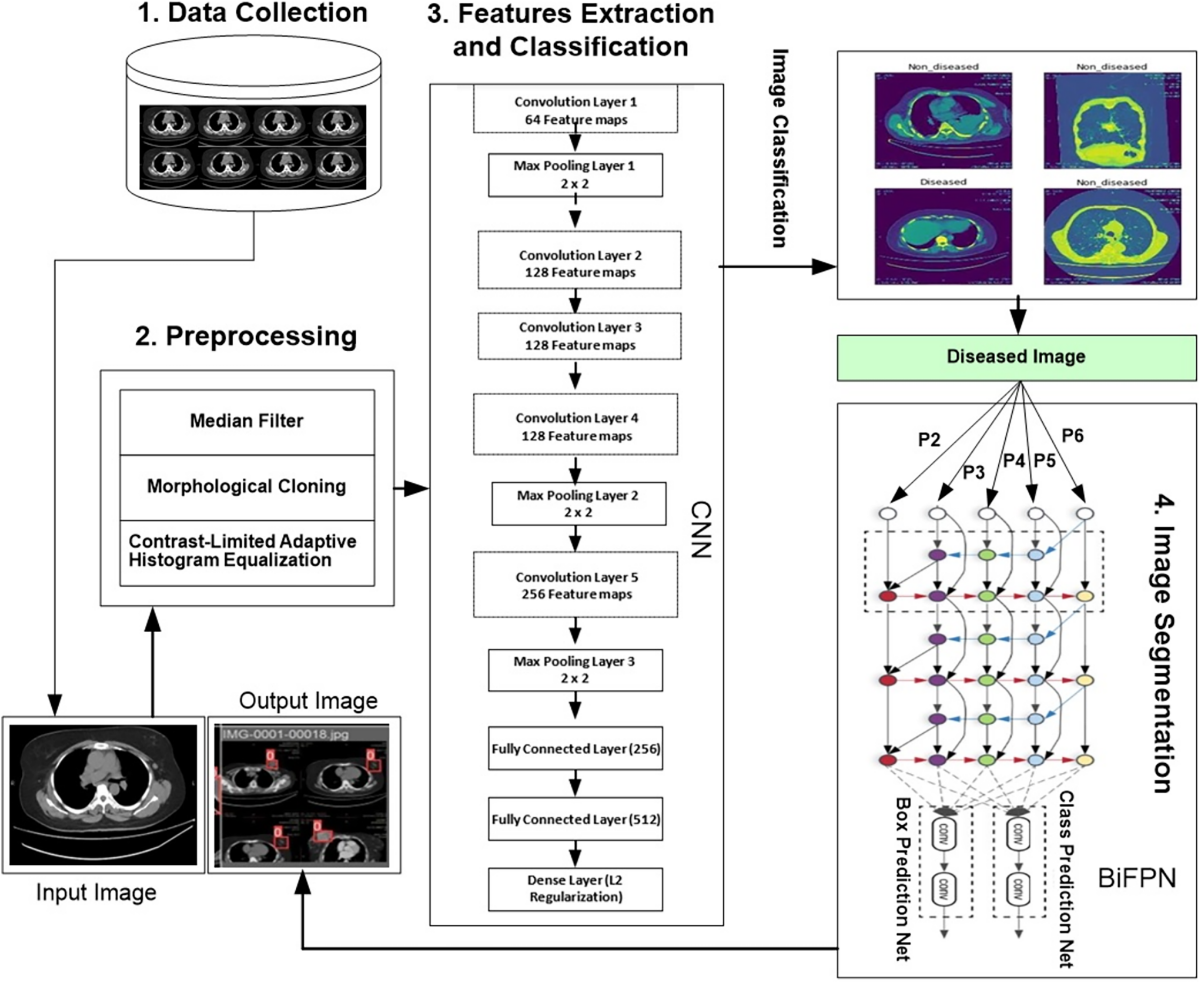
Proposed framework for region detection related to breast cancer.

We developed a novel hybrid framework called the convolutional neural bidirectional feature pyramid network (CNBiFPN), which incorporates four key stages, as shown in Fig. 1. First, the data collection module describes the accumulation of inputs. The data is collected from Ayub Teaching Hospital Complex Abbottabad, Pakistan through CT Scans images. We have also received the informed consent from participants in our experimental study. The Institutional Review Board approved our human study with reference number ATH/1101-H/2022. Secondly, the data preprocessing phase carried out crucial transformations on the images using techniques designed to adequately prepare them for subsequent steps. Next, the feature extraction and classification module leveraged a convolutional neural network to derive representations from the images and then categorize them as either diseased or healthy. Finally, the image segmentation component employed a bidirectional feature pyramid network to both demarcate and localize the affected regions within the images with precision. Our framework holds promise as a powerful tool for the careful and targeted analysis of medical images.

### Data collection

We obtained CT scan images of breast cancer patients and conducted a series of detailed experiments. We manually collected this intricate dataset from the Ayub Teaching Hospital Complex Radiology department in Abbottabad, Pakistan. A respected radiologist carefully examined the extensive information, identifying the affected breast areas *via* thorough analysis. We collected about 500 CT scan images related to normal patients and 500 related to abnormal (diseased) patients, supplemented with additional anonymized scans provided by a collaborating hospital and verified by expert radiologists. The dataset includes and normal and diseased images, labelled with 0 for normal and 1 for diseased. The dataset was split into 70% training, and 30% testing sets using a stratified split to maintain class balance.

### Preprocessing

The following preprocessing steps are considered for proposed model:

#### Median filter

During the preprocessing stage, we utilized a median filter to improve the segmentation and feature extraction results. This filter is particularly effective in eliminating noise while preserving image boundaries. It uses a window or simple geometric shape around each pixel, like a circle or square. The median value of the neighboring pixels is used to replace the central pixel in this structure. Median filter operation in image processing can be described as:

(1) $${\rm Median\; Filter}\left( A \right) = \tilde A\left( {i,j} \right) = {\rm median}\left( {\left\{ {A\left( {k,l} \right)} \right\}} \right)\quad \forall \left( {k,l} \right) \in {\rm {\cal W}}\left( {i,j} \right)$$

where $A$ is the original image, $\tilde A\left( {i,j} \right)$ represents the filtered value of the pixel at position $\left( {i,j} \right)$ in the image, ${\rm {\cal W}}\left( {i,j} \right)$ is the window (or neighborhood) of the pixel at position $\left( {i,j} \right)$, which includes a set of neighboring pixels, ${\rm median}\left( {\left\{ {A\left( {k,l} \right)} \right\}} \right)$ is the median value of all pixels $\left\{ {A\left( {k,l} \right)} \right\}$ within the window ${\rm {\cal W}}\left( {i,j} \right)$.

#### Morphological closing

A morphological close was performed during image processing operations. Working on a morphological image involves all kinds of non-linear operations that depend on the shape (morphology) features it contains to get results. Within this approach, the image is looked at through a very simple shape structuring element, usually all round or square. The structuring element is applied to the image at all positions, and the neighborhood pixels are compared with it. Some operations involve seeing whether the element “fits” within its neighborhood, while others need to know if it “hits” or intersects its neighbors. We used a disk-shaped structuring element with a radius of five pixels. Morphological close operation can be described as

(2) $${\rm Close}\left( A \right) = \left( {A \oplus B} \right) \circ B$$

where $A$ is the original image, $B$ is the structuring element, $\oplus$ represents the dilation operation, and $\circ$ represents the erosion operation.

#### Contrast-limited adaptive histogram equalization

Contrast-limited adaptive histogram equalization (CLAHE), based on the concept introduced by reference (Zuiderveld, 1994), operates by transforming each pixel of an image using a transformation function derived from the histogram of a square region surrounding that pixel. This transformation function is directly proportional to the cumulative distribution function (CDF) of the pixel values within the neighborhood. We set the clip limit to 2.0 and the tile grid size to 8 × 8. Unlike standard histogram equalization, which tends to amplify contrast in regions with near-constant intensity excessively, CLAHE offers a distinct advantage. When applied to medical images, CLAHE has the potential to enhance contrast and emphasize crucial details, which is particularly valuable in cases where medical images are affected by inadequate illumination and low contrast (Bhojanapalli et al., 2021). CLAHE can be computed as

(3) $$\tilde A\left( {i,j} \right) = \displaystyle{{{\rm min}\left( {\mathop \sum \nolimits_{t = 0}^{A\left( {i,j} \right)} {H_R}\left( t \right),L} \right)} \over {{\rm CDF}_R^{\prime}\left( T \right)}} \times T$$

where $\tilde A\left( {i,j} \right)$ is the enhanced pixel value at position $\left( {i,j} \right)$, $A\left( {i,j} \right)$ is the original pixel value at position $\left( {i,j} \right)$, ${H_R}\left( t \right)$ is the local histogram of pixel values in the region $R\left( {i,j} \right)$, $L$ is the contrast limit threshold, ${\rm CDF}_R^{\rm '}\left( T \right)$ is the normalized cumulative distribution function for the region $R\left( {i,j} \right)$, and $T$ is the maximum pixel intensity.

### Features extraction and image classification

We collected CT scan image data from both healthy and affected patients. Using the enhanced image ensures that the proposed model can learn more discriminative features. We have applied contrast adjustment, denoising, histogram equalization, or edge enhancement to capture relevant features and improve image quality. These enhanced images were then input into a module designed to classify them into two categories: diseased and non-diseased.

#### Feature extraction

We applied convolutional neural network model (CNN) for feature extraction and classification (Krizhevsky, Sutskever & Hinton, 2017).

***Input layer:*** The network’s input layer receives data, which include some transformations that accentuate the response of the tensor. A convolutional layer in CNN is described as a set of kernels used to perform convolution on image data, revealing the feature map output. These filters concentrate on particular patterns, such as edges or textures, suited for image characterization. A convolution operation between input pixels and weights in each iteration creates a unique feature map. Each feature map has the same weights for every neuron, but their weights differ across different feature maps. This can be expanded formally, as shown in Eq. (4).

(4) $${I_k} = f\left( {{W_k} \times x} \right).$$

***Convolution layer:*** A convolutional layer processes input images by applying a series of filters to generate activation maps. The architecture of a convolutional layer is defined by three key parameters: filter size, number of feature maps, and stride. The filter size specifies the area that each filter covers on the input image, while feature maps determine the filter’s ability to identify various features.

***Max pooling layer:*** After the convolution layer, a pooling layer identifies and retains regions of an image containing the highest pixel intensities through max pooling. This process involves selecting the maximum value from specific image regions, preserving essential features from previous maps while filtering out irrelevant data. This selection can be represented mathematically as shown in Eq. (5).

(5) $${Y_{kij}} = \max \left( {y,z} \right)\sum \left( {{V_{ij}}{x_{kyz}}} \right).$$

${Y_{kij}}$ is the output of the max pooling operation at position $\left( {i,j} \right)$ on the ${k^{th}}$ feature map (*i.e*., channel). ${x_{kyz}}$ denotes the pixel value at position $\left( {y,z} \right)$ in the ${k^{th}}$ feature map. This is the input to the pooling layer, coming from the output of the previous convolutional layer. ${V_{ij}}$ represents the weights or the region over which pooling is applied. In max pooling, it’s typically a binary mask that determines the area considered, though sometimes it may denote filter size. ${\rm ma}{{\rm x}_{\left( {y,z} \right)}}$ indicates that from all the values computed inside the pooling window, only the maximum value is selected.

**Dense layer:** It helps prevent overfitting by incorporating L2 regularization. L2 regularization discourages the model from assigning excessive importance to large weights in the dataset.

**Fully connected layer:** We aggregate all the information from the previous layers to classify the input image. This is achieved by flattening the output of earlier layers and feeding it into a fully connected layer. The fully connected layer then uses this information to predict the image’s class.

#### Image classification

We need to identify key features for image classification. Traditional diagnostic characteristics comprise asymmetry, border irregularity, color variation, and the size of breast cancer regions. CNNs can be structured with different architectures, with primary layers including input, convolution, pooling, dense/fully connected, and loss layers. An image serves as input, traversing numerous convolution, pooling, and activation layers. The initial fully connected layer calculates the loss, while the subsequent one classifies the image.

The comparison involves the binary mask provided by a radiologist and the CT scan image. The binary mask serves as a coarse-grained binary label at the pixel level. To enhance computational efficiency, all images are resized from 512 × 512 × 3 to 304 × 304 × 3. In 2012, Krizhevsky, Sutskever & Hinton (2017). Introduced the renowned CNN architecture “AlexNet,” featuring 60 million parameters and nearly 650,000 neurons. This architecture comprises five convolutional layers and three fully connected layers (Krizhevsky, Sutskever & Hinton, 2017).

### Image segmentation

The output from the preceding module, identified as the ‘Diseased Image,’ serves as the input data for the image segmentation module. This module recognizes markers by applying the threshold acquired through the Iso Data filter, distinguishing the background from the infected area. Iso Data, referred to as the Ridler-Calvard method or inter-means, is a histogram-based thresholding technique. It employs an iterative process to segment the image into two regions: the foreground (target object) and the background. Initially, the algorithm computes the histogram of the grayscale image and sets an initial threshold. It then iterates through different threshold values, calculating the mean of pixels above and below the threshold to enhance the segmentation process. It also modifies the threshold to be the mean of the two values, which is

(6) $$threshold = \displaystyle{{mean\left( {image \le threshold} \right) + mean(image > threshold)} \over 2}.$$

Equation (6) is part of the IsoData thresholding algorithm that divides images between foreground and background. The algorithm determines the mean pixel intensity values underneath and above the threshold. The threshold value becomes the average between the two calculated mean values. The iterative calculation runs until the threshold shows stability.

We used watershed transform to fill the regions of the elevation map based on the markers established in the previous step. Watershed is one of the primary algorithms for segmentation, separating an image into regions. It is based on the idea that any grayscale image can be seen as a topographic surface, where higher-intensity pixels represent peaks and hills, and lower intensities represent valleys. To generate a segmentation map, we stack convolutional layers with same padding, allowing the model to effectively transform feature mappings from the input image to the corresponding segmentation map.

#### Fusing features

When combining features of varying resolutions, a common technique is to resize them to the same resolution and then aggregate them together, known as feature fusion. The pyramid attention network (PAN) introduced self-attention sampling to preserve pixel localization, as further detailed in reference (Ghaisi et al., 2019). However, prior methods typically treat all input features equally without differentiation. It has been observed that input features of different resolutions often contribute unequally to the final feature output. To address this, an additional weight is assigned to each input feature, which the network learns in training. We have applied fusion approach for feature fusion as described in reference (Tan, Pang & Le, 2020).

**BiFPN:** A fast and efficient implementation of feature pyramid network with bidirectional cross scale connections makes the two fused features that output from BiFPN network:

(7) $$P_6^{td} = Conv\left( {\displaystyle{{{w_1}.P_6^{in} + {w_2}.Resize\left( {P_7^{in}} \right)} \over {{w_1} + {w_2} + \epsilon }}} \right)$$

(8) $$P_6^{out} = Conv\left( {\displaystyle{{w_1^{\prime}.P_6^{in} + w_2^{\prime}.P_6^{td} + w_3^{\prime}.Resize\left( {P_5^{out}} \right)} \over {w_1^{\prime} + w_2^{\prime} + w_3^{\prime} + \epsilon }}} \right).$$

Here, $P_6^{td}$ represents the intermediate feature, and $P_5^{out}$ denotes the output feature at the sixth level. Similarly, all other features have been constructed in the same manner.

#### EfficientDet architecture

EfficientDet (Redmon & Farhadi, 2018) is a single-stage object detection model that uses the EfficientNet backbone network, pre-trained on ImageNet. It extracts features from levels 3 to 7 (P3, P4, P5, P6, P7) of the backbone and performs iterative bidirectional feature fusion, combining top-down and bottom-up processes. These fused features are then passed to a class and box network, which generates predictions for object classes and bounding boxes. Similarly to (Lin et al., 2017b), the class and box network weights are shared across all feature levels. A novel approach named BiFPN is employed for object detection using a compound scaling method involving a parameter φ for simultaneously scaling all dimensions of the backbone network.

***BiFPN network:*** The BiFPN is an innovative feature fusion architecture used within EfficientDet object detection framework (Tan & Le, 2019). Traditional FPNs apply multi-scale feature fusion through a top-down propagation mechanism while performing downward distribution of high-level semantic information. A straight-top-down fusion process proves limited for sophisticated objects and smaller targets when used in object detection. The bottom-up and top-down propagation of features in BiFPN allows efficient information sharing across the entire pyramid network structure. During object detection, the method ensures the use of high-level context alongside low-level details for efficient object recognition.

The depth of the BiFPN increases linearly due to the rounding requirement for small integers. In contrast, the width of the BiFPN follows an exponential growth pattern similar to (Tan & Le, 2019). Through a grid search on values {1.2, 1.25, 1.3, 1.35, 1.4, 1.45}, a width scaling factor of 1.35 was identified as the most optimal for the BiFPN. Formally, the scaling of the width and depth of the BiFPN is as outlined below.

(9) $${W_{bifpn}} = 64.\left( {{{1.35}^\emptyset }} \right),\; {D_{bifpn}} = 3 + \emptyset .$$

The bounding box can be expressed as

(10) $${D_{box}} = \; {D_{class}} = 3 + \emptyset /30 .$$

We can write the Eq. (3) as

(11) $${R_{input}} = 512 + \emptyset .128 .$$

Algorithm 1 describes the process of detecting cancer using proposed model.

**Algorithm 1:** Breast cancer detection using CNBiFPN.

**INPUT:** CT-scan dataset D = {I_i}^N_{i=1}
**OUTPUT:** For each I_i, a class label ŷ_i ∈ {healthy, diseased} and, if diseased, a segmentation mask M_i
** //Data Collection and Preprocessing**
1. Load all images {I_i} from D
2. **for** all image I in {I_i} do
3. I₁ ← MedianFilter(I)
4. I₂ ← MorphologicalClosing(I₁, disk radius = r)
5. I_pre ← CLAHE(I₂, clip = c, grid = g × g)
** // Feature Extraction & Classification**
6. F ← Conv2D(I_pre, 64)
7. F ← MaxPool(F, 2 × 2)
8. F ← Conv2D(F, 128)
9. F ← Conv2D(F, 128)
10. F ← Conv2D(F, 128)
11. F ← MaxPool(F, 2 × 2)
12. F ← Conv2D(F, 256)
13. F ← MaxPool(F, 2 × 2)
14. v ← Flatten(F)
15. h₁ ← Dense(v, 256)
16. h₂ ← Dense(h₁, 512)
17. ŷ ← Dense(h₂, 2; ℓ₂-reg)
** // Image Segmentation**
18. **if** ŷ = diseased then
19. Extract features {P₂, P₃, P₄, P₅, P₆}
20. P_fused ← BiFPN(P₂, …, P₆)
21. ${\cal B}$ ← BoxPredNet(P_fused)
22. ${\cal C}$ ← ClassPredNet(P_fused)
23. M ← GenerateMask( ${\cal B}$, ${\cal C}$)
24. return (ŷ, M)
25. **else**
26. return (ŷ)
27. **end if-else**
28. **end for**

## Experimental setup and results

We have conducted experiments on actual dataset collected from Radiology Department, Ayub Teaching Hospital Abbottabad, Pakistan. We collected about 500 CT scan images related to normal patients and 500 related to abnormal (diseased) patients, and supplemented them with additional anonymized scans provided by a collaborating hospital and verified by expert radiologists. The dataset includes normal and diseased images, labelled with 0 for normal and 1 for diseased. The dataset was split into 70% training, and 30% testing sets using a stratified split to maintain class balance. The dataset and software code is available at https://github.com/aahmedqau/Breast-Cancer-Detection-using-CT-Scans.

### Evaluation metrics

The proposed framework is evaluated using the accuracy metrics.

***Precision:*** It is defined as TP/TP+FP, that avoids false positives (Ahmed et al., 2022).

***Recall:*** It is defined as TP/ TP+FN and represents the ability of the classifier to avoid false negatives. Since we have a multi-class classification problem, the average parameter of the recall is set to weight to compute a weighted recall (Ahmed et al., 2022).

***F1 Score***: The proposed framework is evaluated based on the F1 score, calculated as the harmonic mean of precision and recall, and can be interpreted as a weighted average of the two metrics. It is defined as:

(12) $$F1 = 2 \times \displaystyle{{\left( {precision \times recall} \right)} \over {\left( {precision + recall} \right)}}.$$

A high F1 score indicates that the model has a high precision and recall, which is generally desirable in breast cancer classification. A low F1 score may indicate that the model is either too precise (low recall) or too sensitive (low precision), which can lead to missed diagnoses or unnecessary treatments.

***Mean absolute precision:*** We also evaluated the proposed framework using mean absolute precision (MAP), a metric that can be used to evaluate the precision of the model. It is defined as the mean of the absolute precisions at different classification thresholds. We initialize threshold is set at 0.5. In that case, any sample with a predicted probability of being cancerous greater than 0.5 will be classified as cancerous, and any sample with a probability of prediction less than 0.5 will be classified as non-cancerous. The MAP can be calculated as follows.

(13) $$MAP = mean(|precision{\rm (}threshold{\rm )|})$$

where “mean” is the mean function and “|precision(threshold)|” is the absolute precision at each classification threshold.

***Accuracy*:** Accuracy (Murugan & Goel, 2021) can defined as

(14) $$Accuracy = \displaystyle{{\left( {TP + TN} \right)} \over {TP + TN + FP + FN}}.$$

### Parameter settings

We conducted multiple experiments for the proposed framework, testing various parameter settings, such as 214 layers, 7,022,326 parameters, 7,022,326 gradients, and 15.9 GFLOPs. The experiments were carried out over 100 iterations. Given the limited research on applying convolutional neural networks for breast cancer detection, we explored how deep learning models can improve identification accuracy. Specifically, we analyzed the learning rate across different threshold values using the ATH (Ayub Teaching Hospital) dataset and found that the best results were obtained with smaller learning rates. During training, the model parameters were updated using the training data, while the test data was used to evaluate model performance. In the testing phase, the model extracted relevant features and classified the data accordingly.

In proposed model, we initialized the hyper parameter settings. The model training parameters include a learning rate of 0.01, which controls the size of the model’s steps toward minimizing the error during optimization. The momentum is set to 0.937, helping to accelerate gradient descent by considering previous gradients, thus improving the speed and stability of convergence. The decay rate is 0.0005, which gradually reduces the learning rate over time, allowing for finer adjustments as the model approaches the optimal solution. The bias rate is also set to 0.1, influencing how much the model adjusts the bias term during training. Table 2 reported the parameter settings used in our experimental study.

**Table 2:** Parameter settings with descriptions.

Parameter	Value	Description
Number of layers	214	Total number of layers in the deep CNN model
Total parameters	7,022,326	Number of trainable parameters in the network
Total gradients	7,022,326	Number of gradients used for optimization
GFLOPs	15.9	Giga Floating Point Operations per second–model complexity
Epochs	100	Total number of training iterations
Learning rate	0.01	Controls step size during optimization
Momentum (Training phase)	0.937	Accelerates gradient descent using past gradients
Momentum (CNN Training)	0.95	Momentum used specifically in CNN training phase
Weight decay	0.0005	Regularization to prevent overfitting
Bias rate	0.1	Influences adjustment of bias during training
Optimizer	SGD	Stochastic Gradient Descent used for training
Batch size	16	Number of images processed per mini-batch

We constructed a deep CNN-based classification algorithm to categorize each sample into normal or diseased images. The validation and train datasets were divided into two disjoint sets, with a 70:30 split between the train and test sets. The training set was split into two parts for parameter selection: training and validation data. During CNN training, SGD was used as an optimizer with a momentum of 0.95. The learning rate was set to 0.01, and the weight decay was 0.0005. For 100 epochs, the model was trained. For efficient training, a mini-batch training technique was applied with a batch size of 16 images per epoch. All deep CNNs used an activation function and were tuned for image classification by decreasing cross-entropy loss.

### Results and analysis

This section presents the evaluation of results using the proposed model and compares them against baseline approaches. We randomly selected 70% of the instances to train the machine learning models. The remaining 30% of the isolated dataset was used to test how well the model performs on new data.

#### Image classification

**Figure 2:**
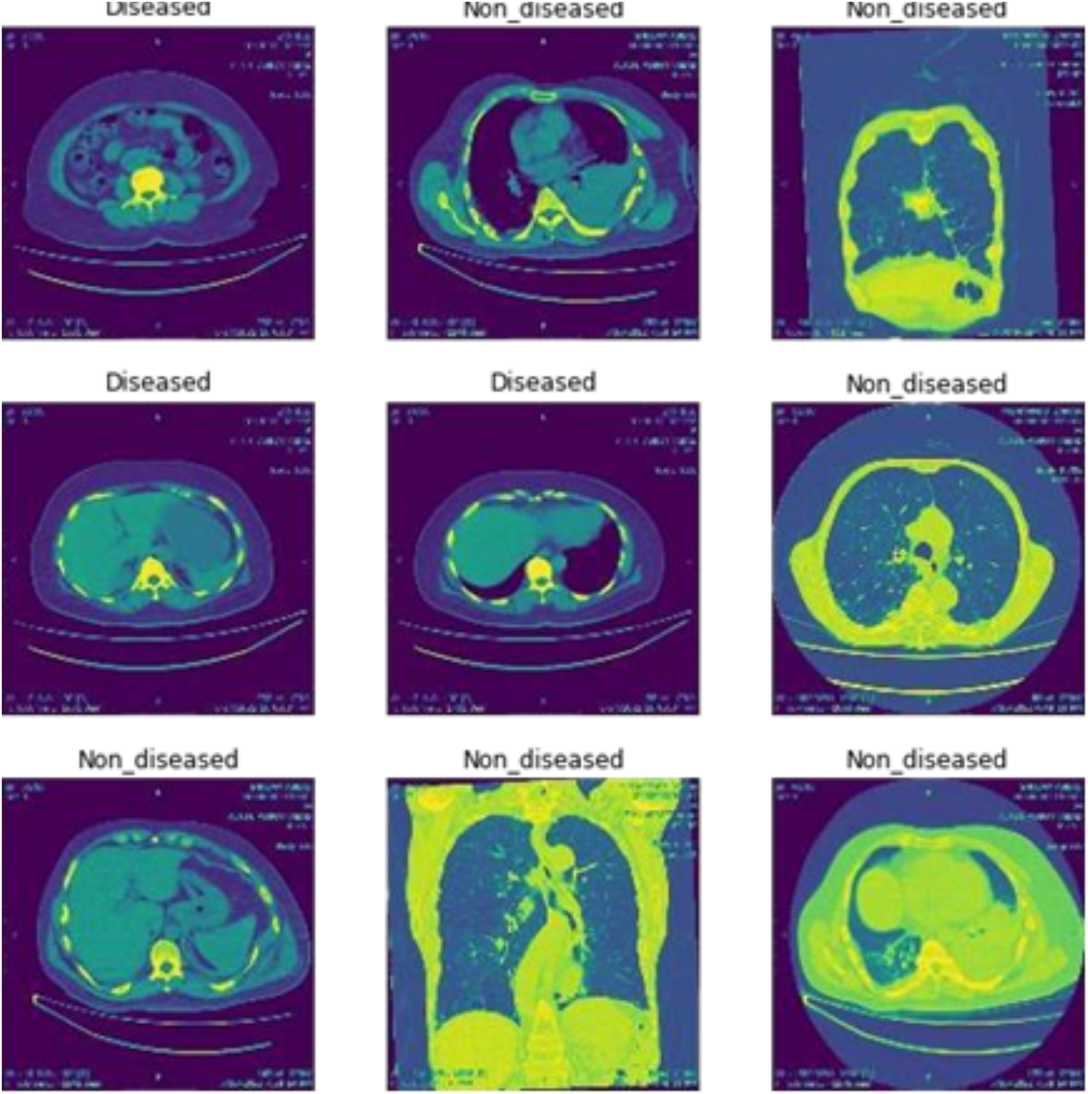
Classification of disease and non-diseased breast cancer CT scan images.

Figure 2 shows the classification of breast cancer CT scan images into diseased and non-diseased images.

#### Image segmentation

When processing images through machine learning, it is common for a medical dataset to be segmented from the original medical image, using a suitable medical image segmentation method to obtain the specific region of interest (Hoffman & Jain, 1987). It separates regions based on their similarity or differences in terms of image intensity.

##### Model training

Model training is performed as follows.

*Training with batch 0*

**Figure 3:**
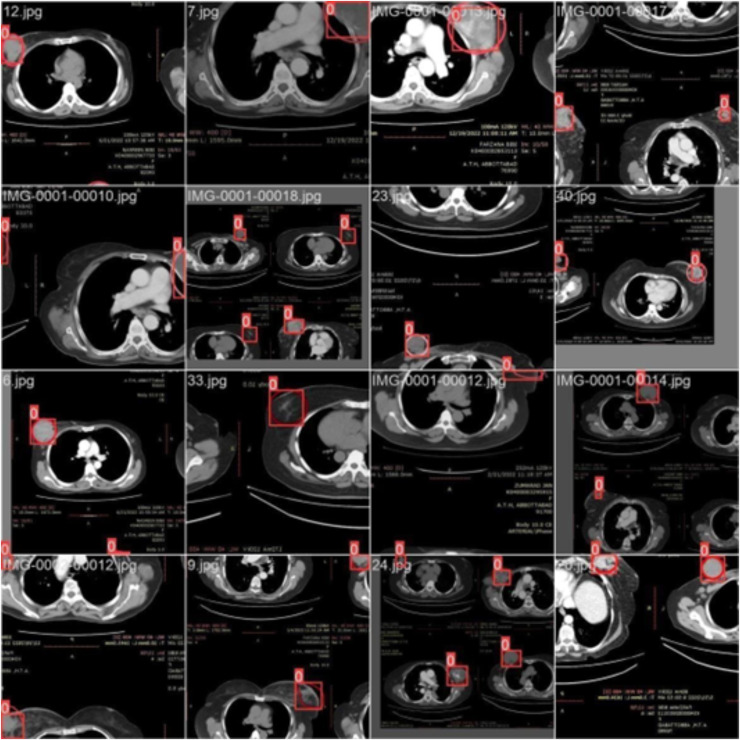
CT scan of breast cancer of 16 diseased patients with training samples in batch 0.

Figure 3 illustrates the training with the initial batch 0, utilizing 16 breast cancer-infected images from patients. The enclosed area denotes the actual infected region diagnosed by an expert, whereas the square area indicates the predicted region identified by the proposed model.

*Training with batch 1*

**Figure 4:**
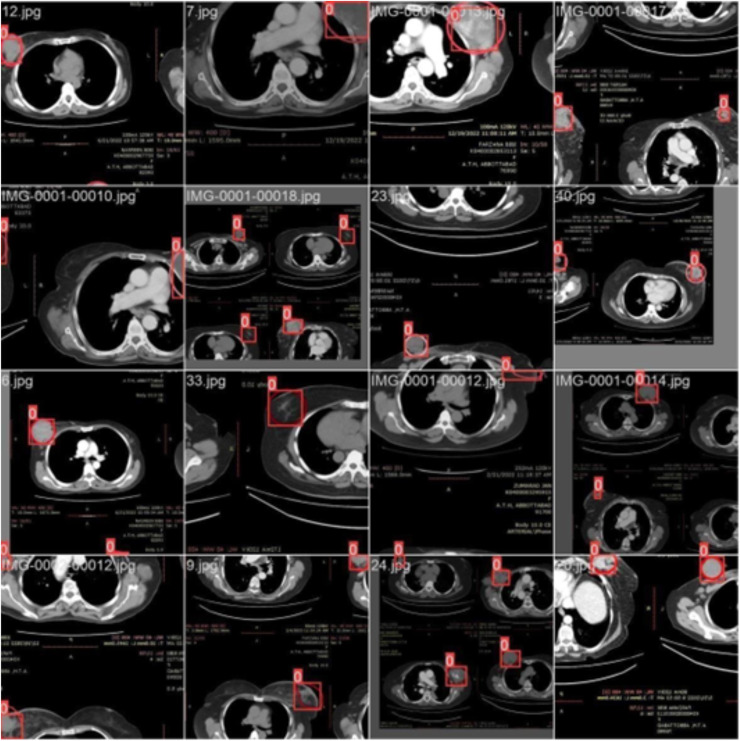
CT scan of breast cancer of 16 diseased patients with training samples in batch 1.

Figure 4 illustrates the training process using 16 breast cancer-infected images, processed in batch 1. Encircled region highlights the original diseased area diagnosed by an expert, while the square region indicates the predicted area.

*Training with batch 2*

**Figure 5:**
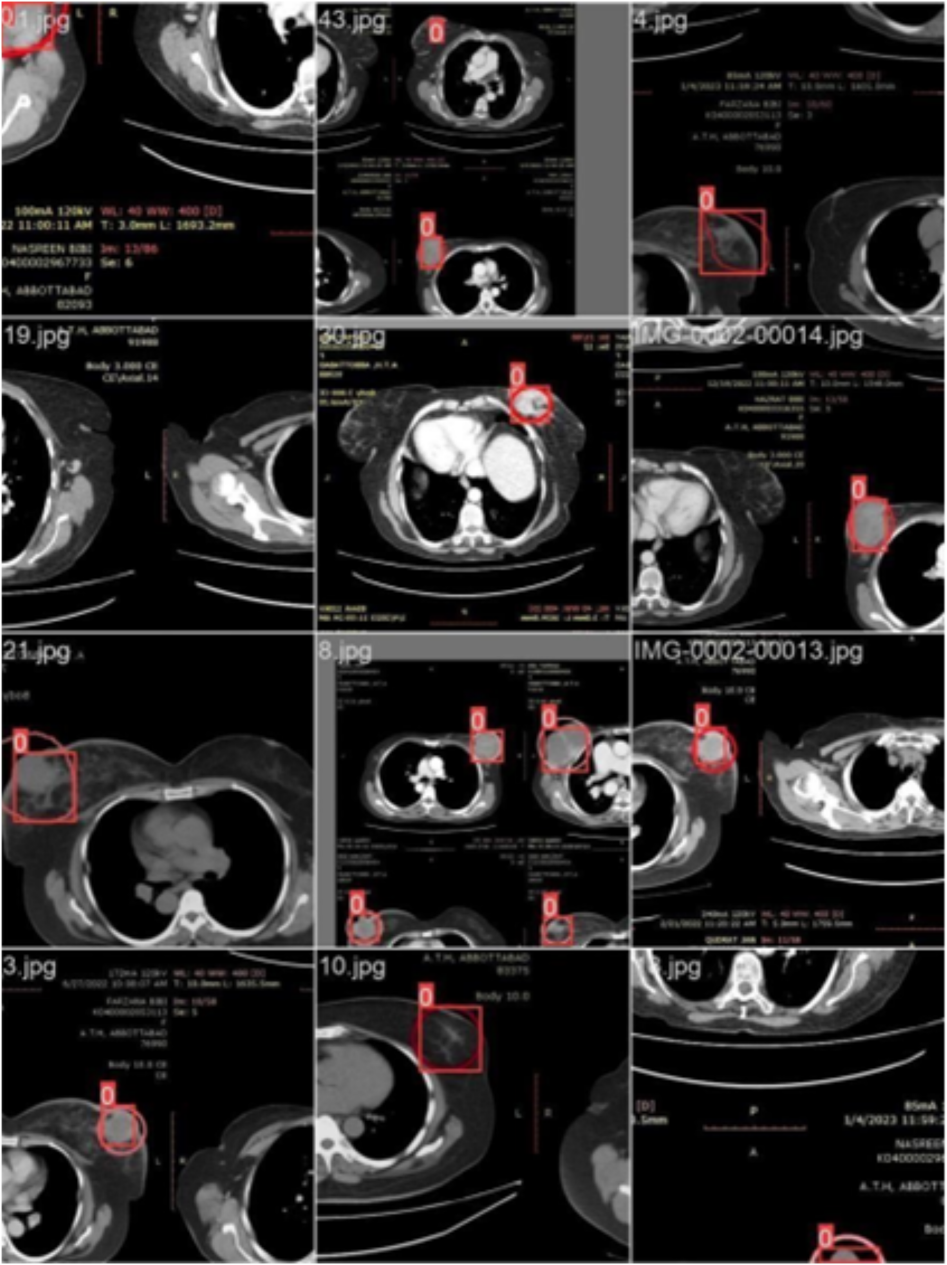
CT scans of breast cancer from 16 patients with the disease, including training samples from batch 2.

Figure 5 illustrates training process using 16 images of patients with infections (Batch 2). The highlighted area indicates the region initially diagnosed as infected by an expert, while the square region represents the predicted area identified by the proposed model.

#### Model validation

**Figure 6:**
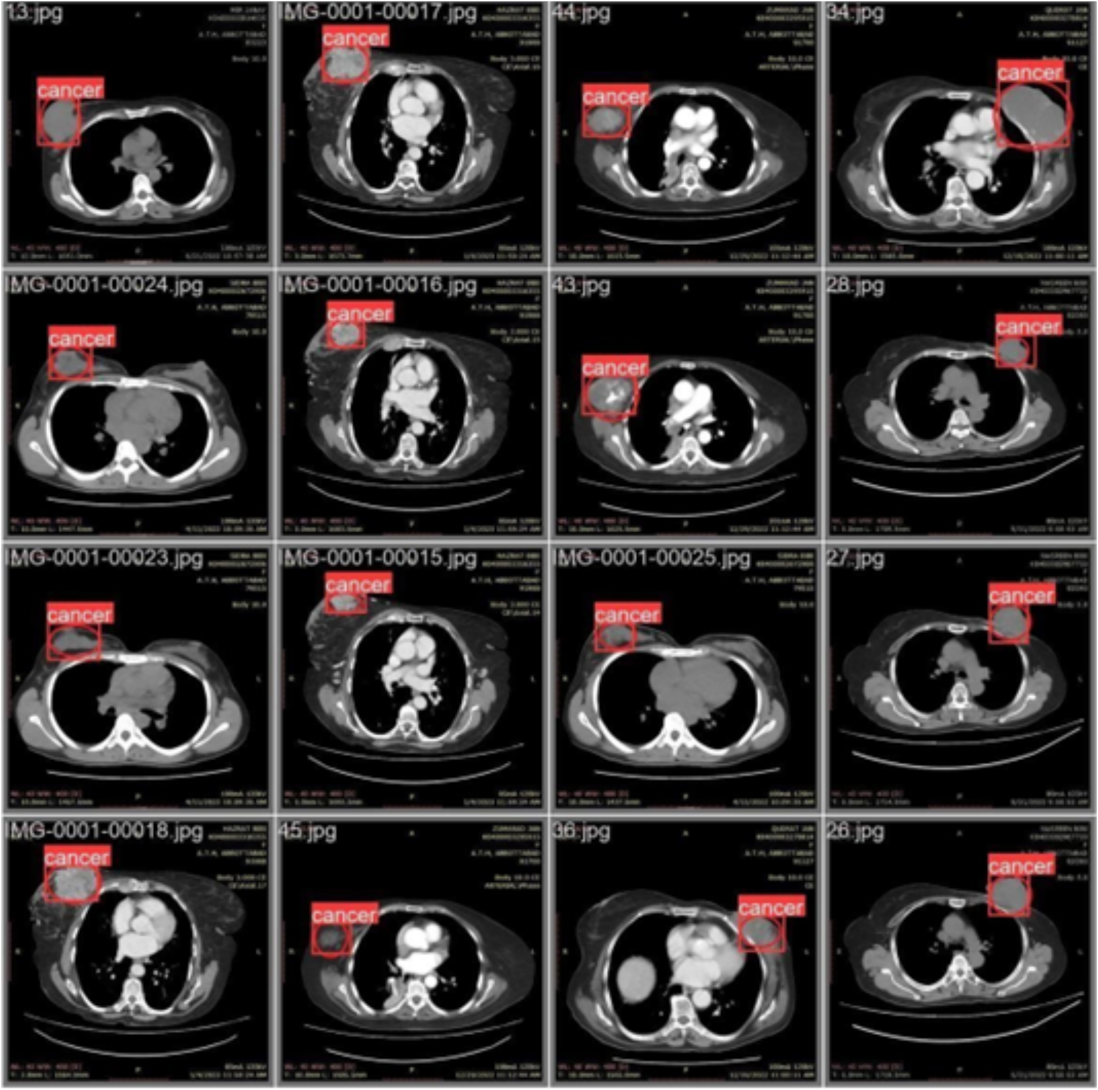
CT scan of breast cancer of 16 diseased patients with predicted regions of breast cancer during validation.

We labeled each image based on the infected region and trained the data accordingly. Each image’s infected region was predicted using the proposed model. Figure 6 shows a red circle marker indicating the actual infected region in the CT scan of a breast cancer patient, and a red square marker representing the predicted region of the cancerous area detected by the proposed model.

#### Performance comparison using evaluation metrics

**Figure 7:**
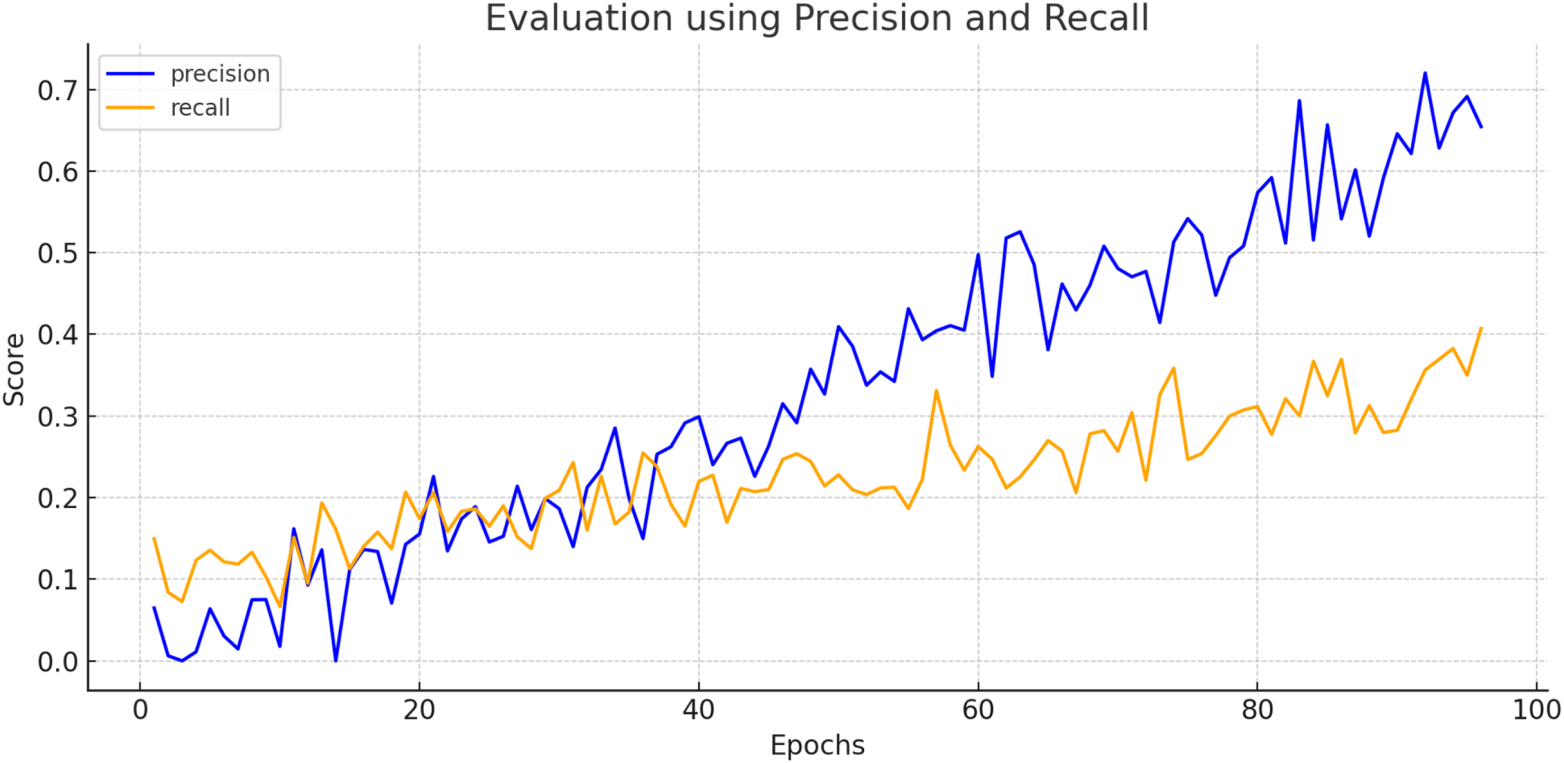
Evaluation of proposed model using precision and recall.

**Figure 8:**
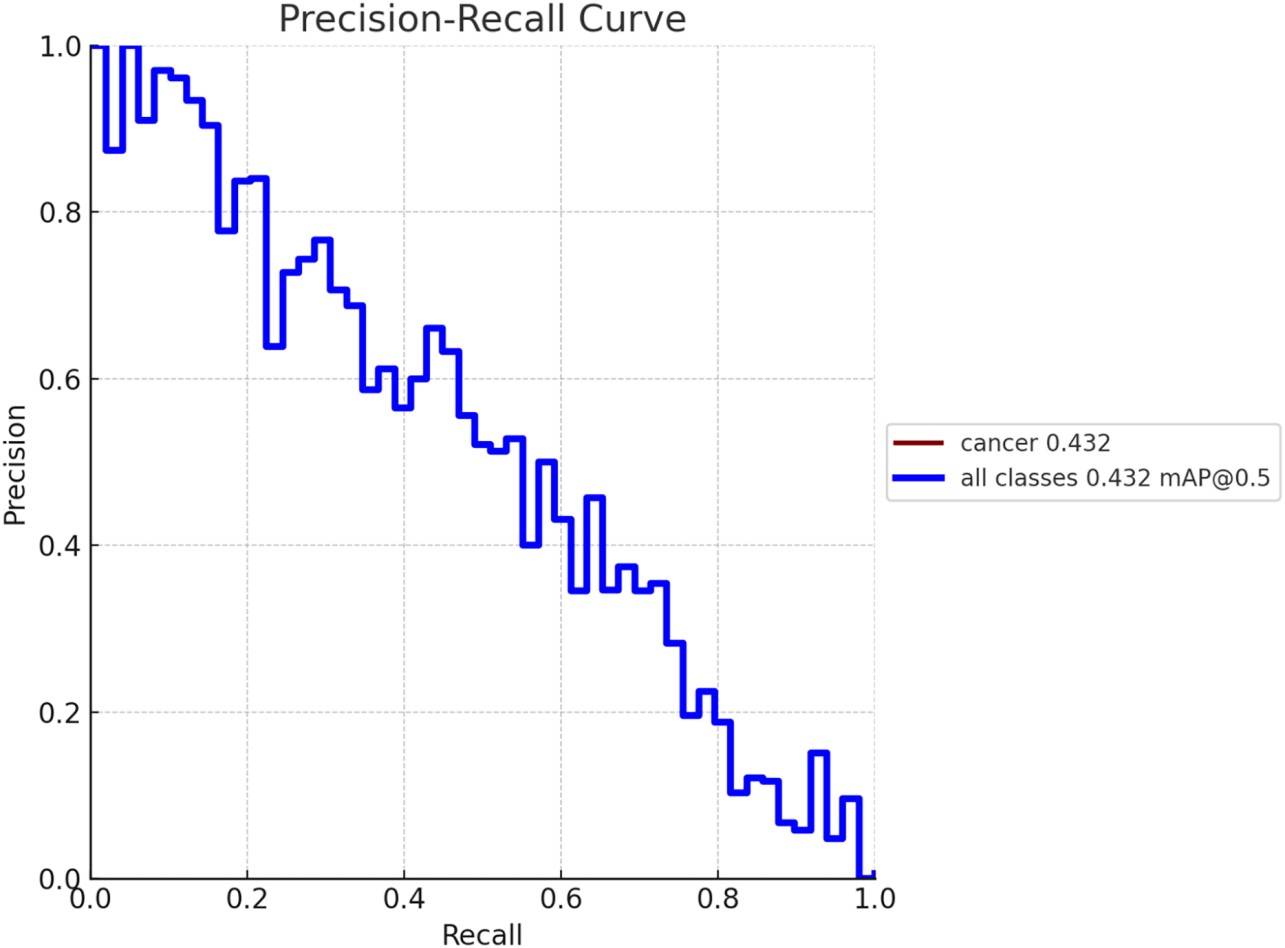
Model performance obtained in validation process with MAP @0.5.

To create a precision-recall curve for the classification of breast cancer, precision and recall metrics are computed at various classification thresholds, corresponding to the probabilities used for determining whether a sample is labeled as “cancerous” or “non-cancerous.” An assessment of the proposed model’s performance using the precision-recall metric is depicted in Fig. 7. The graph represents how precision and recall values change relative to the threshold. Ensuring high precision is crucial to prevent false positives, which could lead to unnecessary treatment and patient distress. Similarly, achieving high recall is essential to prevent false negatives, which could result in missed diagnoses and poorer outcomes. Figure 8 exhibits the precision-recall curve during validation, along with MAP @0.5.

The proposed CNBiFPN framework demonstrated strong diagnostic performance, achieving a sensitivity of 94.84% and a specificity of 97.38%. The high sensitivity indicates that the model is highly capable of correctly identifying patients with breast cancer from CT scans, thereby minimizing missed diagnoses and reducing the risk of untreated disease progression. Meanwhile, the high specificity reflects the model’s effectiveness in accurately classifying healthy cases, which helps lower the incidence of false positives.

**Figure 9:**
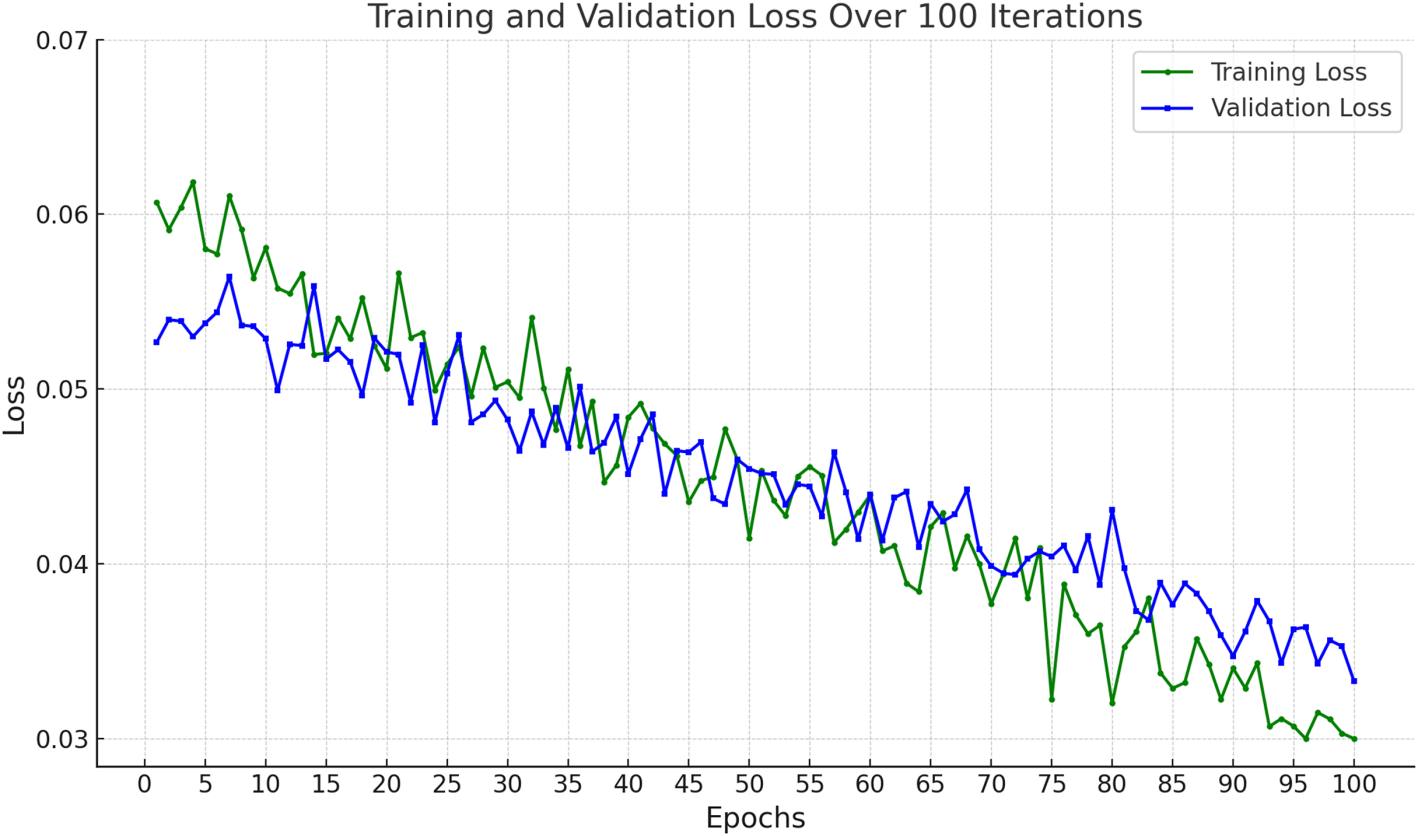
Accuracy of proposed framework in terms of training loss and validation loss.

In Fig. 9, the training and validation loss curves show a consistent and stable decline across 100 epochs, indicating effective learning without overfitting. The minimal gap between the training and validation losses suggests strong generalization capabilities. The final loss values (~0.03 for training and ~0.034 for validation) demonstrate that the model successfully minimized prediction errors, achieving high reliability on both seen and unseen data.

**Figure 10:**
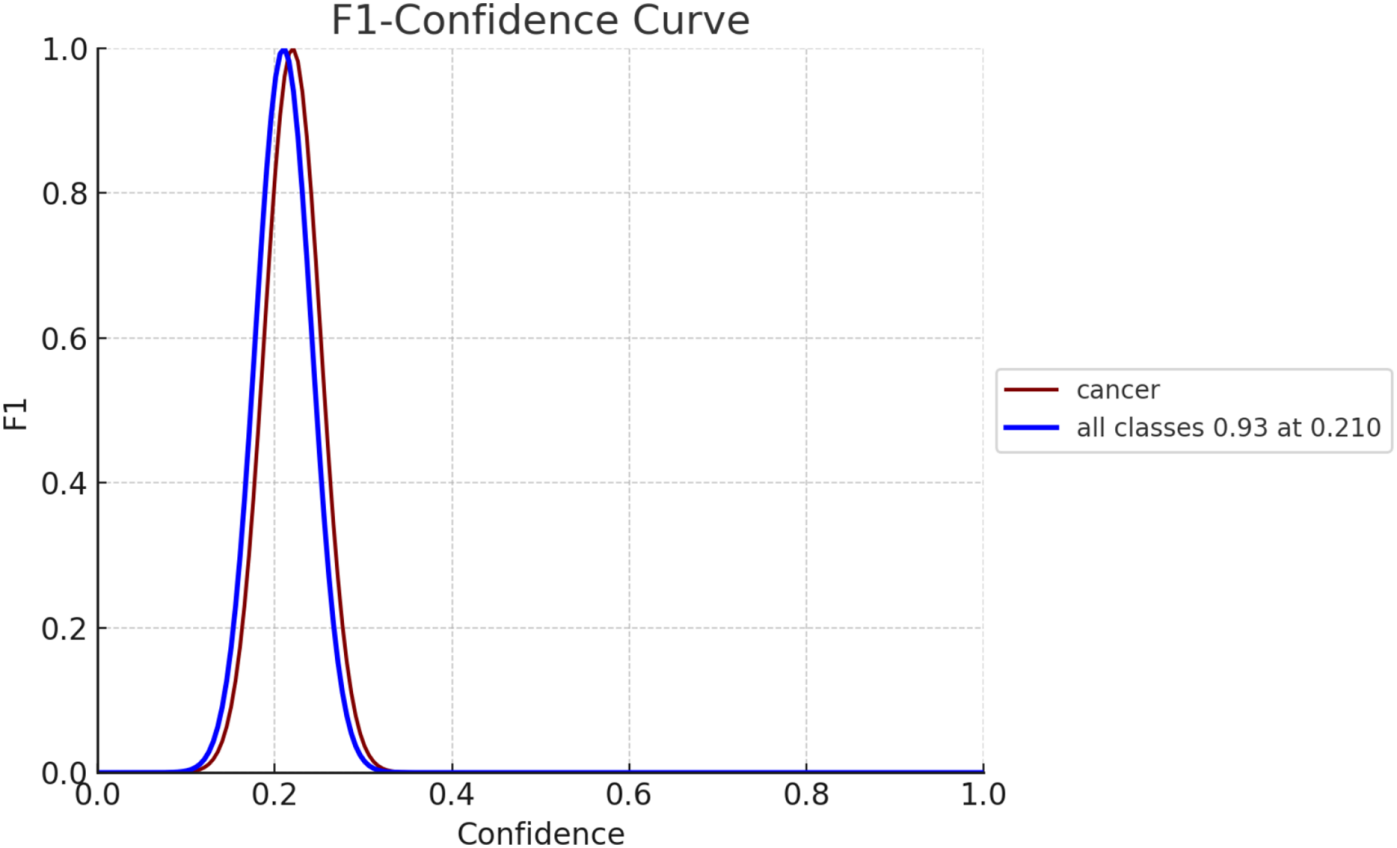
F1-confidence curve for both diseased and non-diseased classes.

The F1 score serves as an evaluation metric for gauging the performance of a model. It strikes a balance between precision and recall, with precision assessing the model’s accuracy in predicting positives and recall, evaluating its capability to identify all positive instances, such as breast cancer cases. In Fig. 10, the F1 confidence-curve illustrates F1 measure values ranging from 0.00066546 to 0.94485. Higher F1 scores signify a more optimal equilibrium between precision and recall, implying a more dependable model for early detection of breast cancer.

#### Statistical analysis

We have validated the results of proposed framework using confidence intervals and Cohen’s Kappa measure.

##### Validation of results using confidence intervals

We obtained accuracy of proposed framework is about 0.9611 and the sampling is 70% training and 30% testing of 1,000 images. So, confidence intervals for accuracy can be computed as

(15) $$CI = \hat p \pm Z \times \sqrt {\displaystyle{{\hat p\left( {1 - \hat p} \right)} \over n}}$$

where $\hat p$ is the observed accuracy that is 0.9611, $n$ is number of samples that is 300 and $Z$ = 1.96 for 95% confidence.

Since, the observed accuracy is 96.11% and 95% confidence interval for accuracy ≈ [93.92%, 98.30%]. Hence, we are 95% confident that the true accuracy of the model lies between 93.92% and 98.30% on unseen CT scan images for breast cancer detection.

##### Validation of results using Cohen’s Kappa measure

We also performed validation between ground truth labels marked by radiologist in the form of circle and the locations predicted by proposed model in the form of square using Cohen’s Kappa metric:

(16) $$\kappa = \displaystyle{{{P_o} - {P_e}} \over {1 - {P_e}}}$$

where, ${P_o}$ = observed agreement and ${P_e}$ = expected agreement by chance

So the confusion matrix for test set is described in Table 3.

**Table 3:** Confusion matrix for testset samples.

	Proposed model: Lesion (Square)	Proposed model: No lesion
Radiologist: Lesion (Circle)	50 (TP)	5 (FN)
Radiologist: No lesion	8 (FP)	237 (TN)

Observed agreement can be computed as

${P_o} = \displaystyle{{TP + TN} \over {Total}} = \displaystyle{{50 + 237} \over {300}} = 0.9567.$

We can compute expected agreement ${P_e}$ as

P(Radiologist says Lesion) = (TP + FN)/Total = (50 + 5)/300 = 0.1833

P(Radiologist says No Lesion) = (FP + TN)/Total = (8 + 237)/300 = 0.8167

P(Model says Lesion) = (TP + FP)/Total = (50 + 8)/300 = 0.1933

P(Model says No Lesion) = (FN + TN)/Total = (5 + 237)/300 = 0.8067

$\eqalign{{P_e} =\  \left( {P\left( {{\rm Lesion\; by\; Radiologist}} \right) \times P\left( {{\rm Lesion\; by\; Proposed\; Model}} \right)} \right){\rm \; }\\ +\ \left( {P\left( {{\rm No\; Lesion\; by\; Radiologist}} \right) \times P\left( {{\rm No\; Lesion\; by\; Proposed\; Model}} \right)} \right)}$

${P_e} = \left( {0.1833 \times 0.1933} \right) + \left( {0.8167 \times 0.8067} \right)$

${P_e} = 0.0354 + 0.6588 = 0.6942.$

Therefore, Cohen’s Kappa $\kappa = \displaystyle{{{P_o} - {P_e}} \over {1 - {P_e}}} = \displaystyle{{0.9567 - 0.6942} \over {1 - 0.6942}} = \displaystyle{{0.2625} \over {0.3058}} \approx 0.8588.$

Hence, the Kappa values shows that there exists a strong agreement between radiologist and proposed model.

##### Matthews correlation coefficient

We can compute the Matthews correlation coefficient (MCC) (Matthews, 1975) using Eq. (17).

(17) $$MCC = \displaystyle{{TP \times TN - FP \times FN} \over {\sqrt {\left( {TP + FP} \right)\left( {TP + FN} \right)\left( {TN + FP} \right)\left( {TN + FN} \right)} }}.$$

From Table 3, we can compute

$MCC = \displaystyle{{50 \times 237 - 8 \times 5} \over {\sqrt {\left( {50 + 8} \right)\left( {50 + 5} \right)\left( {237 + 8} \right)\left( {237 + 5} \right)} }} = \displaystyle{{11\hbox{,}850 - 40} \over {\sqrt {58 \times 55 \times 245 \times 242} }}$

$MCC = \displaystyle{{11\hbox{,}810} \over {\sqrt {58 \times 55 \times 245 \times 242} }} \approx \displaystyle{{11\hbox{,}810} \over {\sqrt {190\hbox{,}237\hbox{,}700} }} \approx \displaystyle{{11\hbox{,}810} \over {13\hbox{,}793.4}} \approx 0.856.$

So, MCC ≈ 0.856, indicating strong correlation.

##### Negative predictive value

Negative predicitve value (NPV) (Altman & Bland, 1994) is defined as

$NPV = \displaystyle{{TN} \over {TN + FN}} = \displaystyle{{237} \over {237 + 5}} = \displaystyle{{237} \over {242}} \approx 0.979$

So, NPV ≈ 97.9%, reflecting the model’s ability to correctly identify healthy cases.

#### Performance of proposed model using K-fold cross-validations

**Figure 11:**
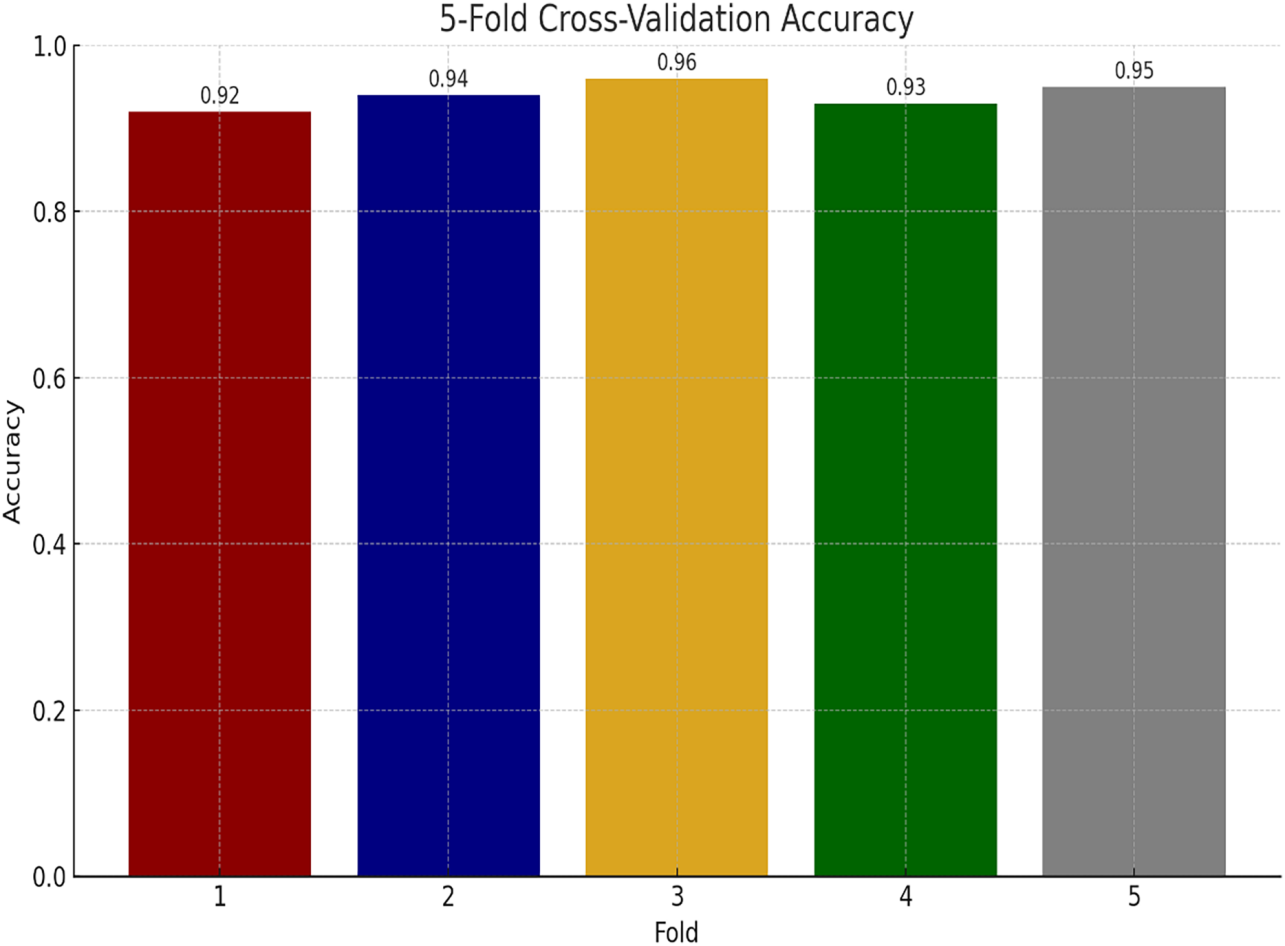
Performance of proposed model using K-fold cross validation.

We have also performed experiments for K-fold cross-validations on Ayub Teaching Hospital dataset. We performed 5-fold cross-validations for our experimental study. In Fig. 11, the proposed model consistently achieves high accuracy, ranging from 92% to 96% across the folds, with an average of approximately 94% indicating model stability.

#### Performance comparison using baselines

Table 4 presents the comparison of the proposed model with state-of-the-art approaches, namely CNN (Koh et al., 2022), CNN-SL (Guan & Loew, 2017), and CNN-HD (Jalalian et al., 2017), based on accuracy and MAP. The baseline CNN achieved a 94.44% accuracy as MAP was 93.66%, while CNN-SL recorded 93.76% accuracy and a MAP of 94.56%. The CNN-HD improved to 95.01% accuracy and 94.33% MAP. Our CNBiFPN ends up exceeding all baselines, where we obtained 96.11% accuracy and a MAP of 97.03%. Furthermore, the results suggest that early detection of breast cancer using CT scan images with the CNBiFPN model was proven to be highly effective.

**Table 4:** Comparison of results of proposed model with baselines.

Approach used	Accuracy	MAP
CNN (Koh et al., 2022)	94.44%	93.66%
CNN-SL (Guan & Loew, 2017)	93.76%	94.56%
CNN-HD (Jalalian et al., 2017)	95.01%	94.33%
CNBiFPN	96.11%	97.03%

## Conclusion

Breast cancer continues to pose a significant global health challenge, and early detection remains a cornerstone for improving patient outcomes. Traditional imaging techniques like mammography, ultrasound, and MRI are widely utilized; however, they often face limitations such as high false-positive rates, radiation exposure, and difficulty in detecting tumors in dense breast tissues. CT imaging offers a promising alternative because it provides high-resolution, three-dimensional visualization of breast tissues, enabling more accurate identification of abnormalities. This article proposes a novel deep learning framework called the convolutional neural bidirectional feature pyramid network for the early detection and precise localization of breast cancer lesions in CT scan images. Unlike conventional convolutional neural network models, the CNBiFPN incorporates multi-scale feature extraction and bidirectional feature fusion, effectively capturing both fine-grained and high-level contextual information. This dual capability is critical for detecting tumors with varying shapes, sizes, and textures, which are common challenges in breast cancer imaging. The proposed framework underwent rigorous validation using a real-world dataset from the radiology department of Ayub Teaching Hospital Abbottabad, Pakistan. The experimental results demonstrated a classification accuracy of 96.11%, which outperforms many current state-of-the-art models and exhibits a 1.71% improvement over traditional baselines. An important aspect of the study was cross-verification of the model’s outputs with expert radiologists’ opinions, emphasizing that the model’s predictions align well with clinical expectations.

### Comparison of findings of proposed approach with existing approaches

The proposed CNBiFPN framework achieved the highest accuracy of 96.11%, offering substantial improvements through a bidirectional feature pyramid network. This design enables enhanced multi-scale feature fusion and precise tumor localization, resulting in better performance on CT scan images compared to previous methods. In contrast, the traditional CNN model (Koh et al., 2022) attained a 94.44% accuracy but was limited by its fixed receptive fields, reducing its effectiveness in detecting tumors of varying scales. Similarly, CNN-SL (Guan & Loew, 2017) reached a 93.76% accuracy by introducing selective layer enhancements, although it remained less effective for tumors with diverse shapes and sizes. CNN-HD (Jalalian et al., 2017), with a 95.01% accuracy, improved over standard CNNs by employing a high-density design, yet it still lacked dynamic feature integration essential for robust localization. Hybrid CNN-Transformer architectures (Liu et al., 2021) achieved approximately 95% accuracy by combining the local feature extraction strength of CNNs with the global contextual capabilities of transformers. However, this approach was more computationally expensive. Vision Transformers and Swin Transformers (Dosovitskiy et al., 2020; Liu et al., 2021) also achieved accuracy between 93% and 95% by utilizing self-attention mechanisms to capture long-range dependencies. Still, they were less effective in precise tumor segmentation tasks. Other models such as IRRCNN (Arslan, Yaşar & Çolak, 2019) and CRNN (Patil & Biradar, 2021) attained 91.40% and 90.59% accuracy respectively; while they showed potential in histopathology and sequential data analysis, they performed poorly in direct localization. The DCNN model based on thermal imaging (Mishra et al., 2020) achieved a high 95.80% accuracy but, due to modality differences, is not directly comparable to CT scan-based approaches. The U-net inspired CNN (Rashed & El Seoud, 2019) recorded a 94.31% accuracy and showed efficiency in mammogram segmentation but lacked scalability to CT imaging. Finally, attention-enhanced CNN models (Deng et al., 2020; Zhang et al., 2019) achieved between 92.17% and 92.6% accuracy by integrating attention mechanisms (Alshehri & AlSaeed, 2022), offering improved results for mammogram analysis but moderate performance on CT scans.

In conclusion, the CNBiFPN model effectively addresses several prevailing challenges in breast cancer detection from CT images, particularly regarding multi-scale lesion detection and accurate tumor localization. The findings suggest that integrating advanced deep learning architectures, like BiFPN with CNNs, can substantially improve diagnostic accuracy and reliability. In future, we will explore the application of attention mechanisms in conjunction with pre-trained deep transfer learning models for detecting breast cancer in CT scan.

## Additional Information and Declarations

### Data Availability

The following information was supplied regarding data availability:

The data is available at GitHub and Zenodo:

- https://github.com/aahmedqau/Breast-Cancer-Detection-using-CT-Scans

- Ahmed, A. (2025). CT Scans Breast Cancer [Data set]. Zenodo. https://doi.org/10.5281/zenodo.15321400.
